# Association between coronavirus 2 infection and preeclampsia among unvaccinated women

**DOI:** 10.61622/rbgo/2025rbgo36

**Published:** 2025-07-15

**Authors:** Mariliza Henrique, Luis Carlos Machado, Carla Gianna Luppi, Vanessa de Oliveira Maciel, Caio Carrete Mazzei, Jessica Macedo Lemos, Marcelo Luis Steiner

**Affiliations:** 1 Hospital da Mulher de São Bernardo do Campo SP Brazil Hospital da Mulher de São Bernardo do Campo, SP, Brazil.; 2 CRT/IST-AIDS Secretaria de Saúde do Estado de São Paulo São Paulo SP Brazil CRT/IST-AIDS Secretaria de Saúde do Estado de São Paulo, São Paulo, SP, Brazil.; 3 Universidade de São Paulo São Paulo SP Brazil Universidade de São Paulo, São Paulo, SP, Brazil.; 4 Universidade Federal de São Paulo São Paulo SP Brazil Universidade Federal de São Paulo, São Paulo, SP, Brazil.; 5 Faculdade de Medicina do ABC Santo André SP Brazil Faculdade de Medicina do ABC, Santo André, SP, Brazil.

**Keywords:** SARS-COV-2, COVID-19, Coronavirus infections, Pre-eclampsia, Hypertension, Pregnancy, Pregnancy trimester, first

## Abstract

**Objective:**

To investigate the association between severe acute respiratory syndrome coronavirus 2 (SARS-CoV-2/COVID-19) infection and preeclampsia (PE); to verify whether the strength of the association differs according to the infection onset (trimester of pregnancy).

**Methods:**

Retrospective cross-sectional study. Included women giving birth at a public hospital in Brazil from July 2020 to January 2021. All women were offered testing for COVID-19 during birth; they were also offered to test during prenatal care if symptomatics or contactants. Excluded women not tested. Compared the frequency of PE as well as of PE superimposed to chronic hypertension (PESCH) in women with versus without infection. Associations were accessed using bivariate and multivariable logistic regression analysis.

**Results:**

Among 1,575 women included, 288 (18.3%) had infection, 53 (3.4%) had PE, and 32 (2.1%) had PESCH. In univariate analysis, infection was significantly associated with PE, but not with PESCH. We then considered only PE as the outcome. The multivariable model included PE, infection, primigravida, fewer than seven prenatal visits. We found association between infection and PE, adjusted odds ratio 2.1; p=0.017. Women infected in the first trimester had a higher frequency of PE than those with infections in the second/third trimester, suggesting a temporal sequence, but the difference wasn't significant (p=0.054).

**Conclusion:**

Our data suggests association between SARS-CoV-2 infection and PE without chronic hypertension. The greater frequency of PE in women who had infection in the first trimester suggests a temporal sequence, but the small numbers are a limitation. Studies with larger samples are welcome.

## Introduction

Coronavirus disease 2019 (COVID-19) has affected about 660 million people and caused about 6.7 million deaths worldwide. Certain groups of individuals have a higher risk of developing severe symptoms, including older adults, black people, and those with obesity, hypertension, or diabetes.^(1)^ Coronavirus 2 (SARS-CoV-2) infection does not seem to be more common in pregnant women than it is in women in the general population. However, this group seems to be at greater risk of serious symptoms. Most pregnant women with COVID-19 have mild symptoms or are asymptomatic, but a relatively high proportion are admitted to intensive care units and need mechanical ventilation.^(2)^ The incidence of adverse outcomes in pregnant women with a SARS-CoV-2 infection has been reported in case series and reviews of case series.^(3,4)^ In cohort studies and meta-analyses of cohort studies in which the incidence of adverse outcomes in such women was analyzed, preeclampsia (PE) or hypertensive disorders of pregnancy was significantly more common in pregnant women with a SARS-CoV-2 infection than that in pregnant women without such an infection.^(5-10)^

One might reasonably expect that COVID-19 increases the risk of PE. Both diseases share common features, such as endothelial damage, imbalance of vasoactive peptides, antiangiogenesis, hypoxia, inflammatory signaling and increased coagulation.^(11,12)^ A "PE-like syndrome" has been described in pregnant women infected with SARS-CoV-2.^(13,14)^ This syndrome has clinical features and yields laboratory test results similar to serious PE, but some women recovered completely before birth once the infection resolves, which is not expected with "true" PE. PE-like syndrome and true PE may be distinguished by the ratio of the antiangiogenic soluble fms-like tyrosine kinase 1 and the proangiogenic placental growth factor, although some consider the strategy unfeasible.^(13,15-17)^

Some data suggest that the reported association between SARS-CoV-2 infection and PE may actually be an association between SARS-CoV-2 infection and PE-like syndrome.^(18,19)^ Khalil et al.^(20)^ recently discussed the possibility of establishing a cause-effect relationship between SARS-CoV-2 infection and PE. Using the nine criteria proposed in 1965 by Sir Austin Bradford Hill,^(21)^ they deemed the research published up to that time lacking in terms of temporal sequence. Later, Hughes et al.^(10)^ reported an association of SARS-CoV-2 infection before or at 28 weeks of pregnancy with hypertensive disorders of pregnancy and other adverse outcomes of pregnancy. A majority of cases of hypertensive disorders occurred in the third trimester. These results may be considered evidence of a temporal sequence. Papageorghiou et al.^(7)^ compared women who had PE and later developed Covid-19 with women who developed Covid-19 not preceded by PE. They discovered that women who had PE developed Covid-19 significantly earlier than those who did not have PE.^(7)^ They concluded that PE increases the risk of Covid-19 rather than that Covid-19 increases the risk of PE.

The objectives of the present study were as follows: to assess the association of SARS-CoV-2 infection during pregnancy with the development of PE; to determine whether such an association differed according to the time of pregnancy in which infection occurred; and to determine, within the group of women with a SARS-CoV-2 infection, whether being symptomatic increases the risk for development of PE. Our hypothesis was that SARS-CoV-2 infection increases the risk of PE.

## Methods

This retrospective cross-sectional study included women who gave birth at *Hospital Municipal Universitário de São Bernardo do Campo f*rom 1 July 2020 to 31 January 2021. This institution is a public teaching hospital in São Bernardo do Campo, an industrial city located in the metropolitan area of São Paulo, Brazil, and attends an average of 4,000 births a year. All women were offered a Covid-19 test at admission for the birth regardless of having symptoms. Most of them were tested. The tests used were the rapid antigen and/or reverse transcription polymerase chain reaction test (RT-PCR) for SARS-COVID 2, depending on availability on the day of admission. Consequently, the decision to test at admission for birth was not related to the clinical presentation. Asymptomatic women with a positive rapid antigen test result were only considered to have an infection after a subsequent positive RT-PCR test result. Conversely, for women with symptoms suggestive of a SARS-CoV-2 infection, we considered a positive rapid antigen test result sufficient for the diagnosis. A small number of symptomatic women were considered to have an infection, despite negative test results, upon typical computed tomography characteristics of COVID-19 pulmonary involvement.

The hospital loads its medical records an electronic system. Data of interest for the research were extracted from the medical records filled during the admission for birth to build a dataset for this research, and tabulated in an Excel sheet.

The city of São Bernardo do Campo has a primary care network, and most women admitted to give birth in our hospital received prenatal care in this network. On the other hand, as this is the only public maternity hospital in the city, most women who received prenatal care in the network gave birth in our hospital. During the study period, universal screening policies were not in place during prenatal care in the study setting, thus asymptomatic women were not tested during pregnancy. Women were offered COVID-19 testing during prenatal care if they had symptoms suggestive of Covid-19 infection, or if they had contact with infected persons. It was utilized the same tests and in the same manner as it was offered for women admitted to give birth. The finding of a positive test for SARS-CoV-2 infection during prenatal care was not a reason for referring the woman to specialized prenatal care, regardless of having or not a serious form of the disease. Thus, in the absence of other conditions which could classify the woman as having a high risk pregnancy, she continued her prenatal care in the primary care unit. We utilized the results of the prenatal COVID-19 tests to assess the association between SARS-CoV-2 infection and PE when they occurred at different times during pregnancy. We accessed data about prenatal care to verify results of tests for COVID-19, and also for the presence or absence of symptoms. All laboratory tests in the municipality units, except those in the emergency units, are performed in a central laboratory, and the results, including those from emergency units, can be accessed electronically in any of the units.

A diagnosis of PE was made if hypertension developed in the second half of pregnancy and any the following criteria were met: 1) proteinuria (≥300 mg of urinary protein in 24 h or a proteinuria/creatinuria fraction ≥0.3), 2) elevation of liver enzymes and/or thrombocytopenia with hemolysis (complete or "incomplete" HELLP syndrome), 3) acute kidney damage: serum creatinine ≥ 1mg/dl, or 4) symptoms suggestive of central nervous system compromise (sudden headache, visual disturbances, or eclampsia).^(22)^ Unfortunately, we were not able to access the conditions fetal growth restriction and/or placental insufficiency in a reliable manner in our sample. In addition, women with documented chronic hypertension who met any the above criteria, consistent with a diagnosis of superimposed PE, were, by the time of data collection, included in the sample. Women with hypertensive disorders of pregnancy are referred to specialized outpatient prenatal care, but also in this setting, they were offered COVID-19 testing only if they had symptoms of infection or had contact with infected persons, that is, the policy was the same as that of primary care. Maternal age, years of schooling, parity, smoking status, alcohol abuse, illicit drug abuse, chronic hypertension, diabetes (overt or gestational), and the number of prenatal visits were assessed as control variables. Unfortunately, we do not have in our medical records data which allow us to calculate the body mass index or to consider the woman as to have obesity. In addition, we determined the presence of symptoms in patients with a SARS-CoV-2 infection. As some SARS-CoV-2 infections were confirmed before admission for birth, we ascertained whether the infection was diagnosed in the first (up to 13 weeks), second (14 to 26 weeks), or third (27 weeks onward) trimester of pregnancy. We also determined the cesarean rate in women with and those without a SARS-CoV-2 infection. COVID-19 vaccination in Brazil started at the end of January 2021. As we collected data for women giving birth up to and including 31 January 2021, our sample can be considered unvaccinated pregnant women. Data were collected from the patients’ electronic records to build the dataset that was analyzed. Women not tested for COVID-19 were excluded from the study.

The size of the sample was calculated through an estimated prevalence of PE of in the population of 6.7% and a prevalence of PE in the infected population of 12%.^(8,18)^ Assuming significance as a value of p<0.05 and a power of 80%, it would be necessary a sample of 1,200 pregnant women.

Numerical variables are presented as means and standard deviations, and qualitative variables as frequencies and percentages. Normally distributed continuous variables were compared between groups with Student's t-test and other continuous variables were compared with the Mann-Whitney test. Normally distributed and other qualitative variables were compared with the chi-square test and the Fischer exact test, respectively.

We accessed the association between SARS-CoV-2 infection with all cases of PE, as well as separately with PE without chronic hypertension and PE superimposed to chronic hypertension. Also, for those women who were tested during prenatal care, we accessed the association of infection in each of the three trimesters of pregnancy with PE, to verify if there was any difference in this association related to the trimester in which the infection occurred.

Associations were accessed using bivariate and multivariable logistic regression analysis, using odds ratios (ORs) as a measure of effect size. Variables were included in the multivariable model if the p-value was ≤0.20 in the bivariate analysis, in ascending order (forward selection). A p-value 0.05 obtained with the Wald test was considered to indicate the significance of individual coefficients in the model. The data were tabulated in Microsoft Excel (Microsoft Corporation, Redmond, WA, USA), and all statistical analyses were performed using STATA software, version 17.0 (StataCorp LLC, College Station, TX, USA). A p-value <0.05 was considered significant.

Our hypothesis was that SARS-CoV-2 infection increases the risk of PE.

The study was approved by the ethics committee of our institution 4.230.314. It was requested a waiver of informed consent, considering that it is a retrospective study accessing records which were utilized for assistance, and it was approved (*Certificado de Apresentação de Apreciação Ética*: 34735220.8.0000.0082).

## Results

**Figure 1:**
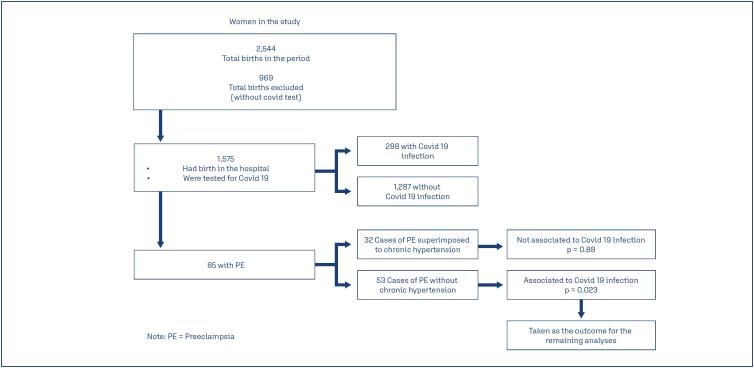
Flowchart of initial analyses

From July 1, 2020 until January 31, 2021, there were 2,544 births in the hospital. After excluding 969 women (38.1%) who were not tested, we had then a sample of 1,575 pregnant women in our study. Among these, 288 (18.3%) had a confirmed SARS-CoV-2 infection, and 85 had a diagnosis of PE (5.5%). Among these 85, 53 (3.4%) had a diagnosis of PE without chronic hypertension, and another 32 (2.1%) had a diagnosis of PE superimposed on chronic hypertension. Considering the whole group of women with PE, that is, women with PE without chronic hypertension ("pure PE") together with those with PE superimposed on chronic hypertension, the association with SARS-CoV infection was not significant, OR=1.44, p=0.16. However, considering the group of PE without chronic hypertension, we found a significant association (OR=1.99; CI95%, p=0.023). Taking only the group of PE superimposed on chronic hypertension, we did not find association with SARS-CoV infection, OR=0.88; p=0.77. Therefore, we decided to consider, for the rest of the analyses, only PE without chronic hypertension as the outcome, and in the tables and the remaining of the text, this group/outcome was designated simply as "PE". This first approach to the analyses is summarized in figure 1.

Table 1 summarizes the associations between maternal variables and PE. The p-value was <0.2 for primigravida, having had fewer than seven prenatal visits, and having 11 or more years of education. The best-fitting logistic regression model included primigravida, and having had fewer than seven prenatal visits. In this model, there remained a significant association between COVID infection and PE; OR 2.1; p=0.017 (Table 2).

**Table 1:** Association between maternal variables and preeclampsia in 1575 women

Variables	Yes (53)	%	No (1,422)	%	p-value
COVID Infection	16/288	(5.6)	37/1,287	(2.9)	0.023
Primigravida	27/593	(4.6)	26/982	(2.7)	0.042
Less than seven prenatal visits	21/363	(5.8)	32/1,212	(2.6)	0.004
11 or more years of schooling	40/1,302	(3.1)	13/271	(4.8)	0.152
Diabetes	1/91	(1.1)	52/1484	(3.5)	0.220
Abuse of Alcohol	1/32	(3.1)	52/1,543	(3.4)	0.939
Abuse of Illicit Drugs	3/48	(6.3)	50/1,527	(3.3)	0.260
Smoking habit	3/139	(2.2)	50/1,436	(3.5)	0.409
AGE					
13-19	5/156	(3.2)	48/1419	(3.4)	0.985
20-29	29/826	(3.5)	24/749	(3.2)	
30-39	17/522	(3.3)	36/1053	(3.4)	
40-49	2/71	(2.8)	51/1504	(3.4)	

YES: proportion of women with preeclampsia within the group with the variable; NO: proportion of women with preeclampsia within the group without the variable; Note: in the first and third columns, denominators change because each line refers to a distinct variable

**Table 2:** Variables associated with preeclampsia in multivariate analysis in 1,575 women

	Adjusted Odds Ratio*	Adjusted confidence interval 95%*	p-value
COVID-19 infection	2.10	1.14 - 3.84	0.017
Primigravida	1.79	1.03 - 3.11	0.038
Less than seven prenatal visits	2.37	1.34 - 4.18	0.003

* Tested for 11 or more years of schooling, besides primigravida and less than seven prenatal visits

Within the SARS-CoV-2-infected group, the frequency of PE was higher among symptomatic (10/106; 9.4%) vs. asymptomatic women (6/182; 3.3%), p=0.028. Cesarean sections were more common in women with a SARS-CoV-2 infection (45%) than it was in women without an infection (36%; p=0.002). An interesting result was that when SARS-CoV-2 infection occurred in the first trimester, the risk of PE was higher compared with the infection that occurred in the second and third trimesters, but the differences were not significant, p=0.054. Among women diagnosed with an infection in the first trimester, 18.8% had PE, compared with 5.8% of those diagnosed with an infection in the second trimester and 4.3% of those diagnosed with it in the third trimester (Table 3).

**Table 3:** Frequency of preeclampsia within the group of 288 COVID infected women, by trimester of pregnancy in which COVID infection was diagnosed

Trimester of pregnancy	First (3-13 weeks)	Second (14-26 weeks)	Third (> 26 weeks)	Total	p-value
Total	3/16 (18.8%)	5/87 (5.8%)	8/185 (4.3%)	16/288	0.054
COVID-19 symptomatic	0/2 (-)	4/31 (12.9%)	6/73 (8.1%)	12/106	0.680
COVID-19 asymptomatic	3/14 (21.3%)	1/56 (1.8%)	2/112 (1.8)	6/182	<0.001

## Discussion

In our study, the diagnosis of PE was confirmed in 5.5% of the sample. It is not much different from the report of Guida et al.^(18)^ In a recent review of the prevalence of PE in Brazil, including ten studies, they found a pooled prevalence of 6.7%, with a 95% confidence interval of 4.5 – 8.6%.

Our data suggests a significant association between SARS-CoV-2 infection and PE, which persisted after multivariate analysis. This association was also clinically relevant, as the frequency of diagnosis of PE in women with a SARS-CoV-2 infection was two times that of women without such an infection. However, we did not find association between COVID-19 infection and PE superimposed to chronic hypertension. It was an unexpected finding, because, in principle, these are also cases of PE. It is not an easy task to find an explanation for this finding. One possible explanation is that the sample is underpowered to detect such an association. Another possible explanation is that PE superimposed to chronic hypertension could have some differences on its pathophysiology, in a way that COVID-19 infection would have less or no influence in the genesis of PE. A less likely but still possible explanation is that the diagnostic criteria for PE superimposed to chronic hypertension that we considered, as stated in Methods, are, besides proteinúria or other abnormalities in the laboratory tests, also the presence of symptoms suggestive of central nervous system compromise like sudden headache, visual alterations, and others. It is possible that some cases of chronic hypertension, without PE, have in some moments, symptoms very similar to these, and then be wrongly diagnosed as PE superimposed to chronic hypertension. The policy of the institution is that, in case of doubt, to favor the diagnosis of PE superimposed, which is the condition with greater risks. But we doubt if that would be the only explanation for this finding. As stated above in this section, if we add the prevalence of PE without chronic hypertension with that of PE superimposed, the result is not much different from the prevalence in the country reported in a recent review, which is even a little higher than that we found (5.5% vs. 6.7%).^(18)^ This suggests that the prevalence of PE superimposed is probably not much lower than the prevalence that we found, what goes against this last hypothesis.

An interesting finding of our study is that SARS-CoV-2 infection in the first and second trimesters of pregnancy yielded a higher frequency of PE than an infection in the third trimester. The differences, however were not significant. Other clinicians reported results that are similar but not identical to ours. Baracy et al.^(5)^ and Rosenbloom et al.^(6)^ reported a significant association of SARS-CoV-2 infection with hypertensive diseases of pregnancy (not only PE). They both conducted secondary analyses, which yielded identical results: the association was significant when infection occurred before 32 weeks of pregnancy, but not thereafter. However, their reports did not mention the time interval between infection and the onset of hypertensive disease. As mentioned in Introduction, Hughes et al.^(10)^ reported a significant association of COVID-19 infection before 28 weeks with hypertensive diseases of pregnancy. The fact that these three studies assessed hypertensive disease of pregnancy, instead of only PE, as the outcome, is not of great importance, as different hypertensive diseases of pregnancy share most pathophysiological determinants. Nobrega et al.^(15)^ stated that their results suggested the possibility that infection early in pregnancy increases the risk of PE, but their results, like ours, were not statistically significant. Our study is unique in that we included data from the first trimester of pregnancy, a period in which PE does not occur. Although not significant in the whole group of infected women (p=0.054), it was significant in the group of asymptomatic infected women (p< 0.001; Table 3). We recognize that the numbers are small, and we cannot affirm, of course, that a significant greater frequency of PE in women infected in the first trimester was found. But we found an interesting trend, and, although the incidence of COVID infection has fortunately decreased, it is still possible that other researchers could find significance with the same approach in larger samples, even if reanalyzing previous data. These findings are in accordance with the proposed pathophysiology of PE: abnormalities in mediators of endothelial function that occur early pregnancy, with vasospasm and ischemia, lay the groundwork for the development of PE in the second half of pregnancy.^(11,12)^ In our view, the results of the present study, taken together with those of the other four studies mentioned in this paragraph, are not a proof, but are suggestive of a temporal sequence between SARS-CoV-2 infection and PE and/or pregnancy-related hypertension.^(5,6,10,15)^ If we consider the existence of a temporal sequence, it does not align with the hypothesis of Sathiya et al.^(14)^ and Rolnik et al.,^(23)^ that the previously reported association between SARS-CoV-2 infection and PE is actually an association between the infection and PE-like syndrome.^(14,23)^ Although, in theory, the inclusion of patients with PE-like syndrome in the group of women with SARS-CoV-2 infection might have contributed to the higher frequency of PE in this group, data suggestive of a time interval between infection and clinical onset of PE goes against the argument of this as the only explanation for the associations found.

Data suggestive of a temporal sequence also offers arguments against the hypothesis propounded by Papageorghiou et al.,^(7)^ that PE is a risk factor for SARS-CoV-2 infection but not vice versa.^(7)^ However, these hypotheses are not necessarily mutually exclusive. In other words, SARS-CoV-2 infection may be a risk factor for PE, as suggested in the present work, and, at the same time, PE may be a risk factor for SARS-CoV-2 infection, as suggested by the work of Papageorghiou et al.^(7)^

In the group of women with SARS-CoV-2 infection, the prevalence of PE was higher among symptomatic compared with asymptomatic women. (symptomatic: 9.4% vs. asymptomatic: 3.3%, p=0.028). On a biological basis, symptomatic infections may yield a greater risk of PE. Metz et al. reported a significantly higher prevalence of PE in critically ill women than in symptomatic or asymptomatic women with SARS-CoV-2 infection.^(24)^ Conde-Agudelo et al. ^(9)^ also reported a higher prevalence of PE in symptomatic than that in asymptomatic women. Cesarean sections are also reportedly more common for women with a SARS-CoV-2 infection, what is in line with our findings.^(8,25)^ Some authors have discussed the reasons for this higher rate of cesareans in women with SARS-CoV-2 infection. Silva et al.,^(26)^ after documenting a significant raise in cesarean section rate during the COVID-19 pandemic in a public hospital in Brazil, verified that most of this increase was due to the raise in the number of cesareans for maternal request. Protection against vertical transmission has been sometimes an argument for choosing a cesarean section, but this protection was not confirmed in clinical research.^(27,28)^ Many times cesarean was indicated because the mothers infected were critically ill; however, cesarean section by itself can worsen the maternal condition.^(16,19)^ In the beginning of the pandemic, the confirmation of SARS-Cov-2 infection was, for many obstetricians, considered by itself an indication for cesarean section.^(29)^ However, there is nowadays a general agreement that this conduct is not supported by scientific evidence.^(27,28)^ We do not have, in our dataset, information about the indications for cesarean section.

In two recent studies, no association was observed between SARS-CoV-2 infection and PE. In one, a case-control study in Brazil, Guida et al.^(30)^ compared 21 women with PE to 183 pregnant women without PE. Their sample of 202 women might not have yielded sufficient power to detect such an association. Tran et al. compared the frequency of PE in 93 pregnant women with confirmed and symptomatic SARS-CoV-2 infection, who gave birth from March to December 2020, with 186 pregnant women who gave birth in the same months a year earlier, in 2019, before the COVID-19 pandemic.^(31)^ The groups did not differ in terms of PE (adjusted OR=3.12; 95% confidence interval=0.39–24.60). The authors themselves stated that their study might have been underpowered for such an analysis.

Our study had many limitations. First, its retrospective nature might have introduced a bias. Second, women not tested for COVID-19 were not included. As stated in Methods, there were two distinct policies for offering the test: during prenatal care (symptomatic women and contactants) and during admission for birth (all women). It should be taken into account the fact that all women were invited to be tested at admission, although some of them were not tested. From those 2,544 women who had birth in the period of the study, 969 (38.1%) were not tested, and consequently, not included. Unfortunately, we do not know the reasons for not testing, but it appears to be more related to administrative aspects than to clinical or epidemiological aspects. Moreover, as data collection finished in January 2021, we do not know whether our results are generalizable to the viral variants that emerged later, such as Delta and Omicron. As the sample consisted of unvaccinated women, we do not know to what extent vaccination would modify our results. Another limitation is that we could not control for body mass index, due to the lack of data in the records, and also, we did not control for maternal diseases other than hypertension and diabetes, because we did not included this information in our dataset. Also, we could not access the variables fetal growth restriction/placental insufficiency as criteria for PE diagnosis. Finally, our conclusions are valid only for PE without chronic hypertension, as we excluded cases of PE superimposed on chronic hypertension from the analyses. The frequency of evolution to superimposed PE among pregnant women with chronic hypertension varies, but there are reports of frequencies as high as 44.7%.^(32)^ Rezk et al.^(33)^ reported 78 cases of PE superimposed to chronic hypertension in a series of 164 women with PE, a frequency of 47.6%. Also in the present study, we had 33 cases of superimposed PE among a total of 85 cases of PE, a proportion of 38.8%. This high frequency could limit the external validity of our conclusions.

The strengths of the study were the sample size, and the finding of a significant two times higher frequency of PE in infected women after adjustment for confounders through multivariable analysis.

The results of this study reveal the importance of prenatal care for pregnant women infected with SARS-CoV-2. Certain clinicians recommend prophylactic aspirin in such cases to prevent PE and other complications of pregnancy.^(34,35)^ However, to date, evidence about the benefit of this intervention is lacking.

## Conclusion

Although more than four years have passed since the beginning of the COVID-19 pandemic, many knowledge gaps about this disease remain, along with the research necessary to fill them.
